# É Hora de Revisitar os Limites da Reserva de Fluxo Fracionada?

**DOI:** 10.36660/abc.20230363

**Published:** 2023-06-28

**Authors:** Fernando Mendes Sant’Anna, Lucas Bonacossa Sant’Anna, Sérgio Lívio Menezes Couceiro

**Affiliations:** 1 Universidade Federal do Rio de Janeiro Macaé RJ Brasil Universidade Federal do Rio de Janeiro - Campus Macaé, Macaé, RJ – Brasil; 2 Serviço de Hemodinâmica Hospital Santa Izabel Cabo Frio RJ Brasil Serviço de Hemodinâmica do Hospital Santa Izabel, Cabo Frio, RJ – Brasil; 3 Fundação Técnico-Educacional Souza Marques Rio de Janeiro RJ Brasil Fundação Técnico-Educacional Souza Marques (FTESM), Rio de Janeiro, RJ – Brasil; 4 Departamento de Cardiologia Hospital Santa Izabel Cabo Frio RJ Brasil Departamento de Cardiologia do Hospital Santa Izabel, Cabo Frio, RJ – Brasil

**Keywords:** Pericárdio, Constrição Patológica, Doença Arterail Coronariana, Intervenção Coronária Percutânea, Reserva Fracionada de Fluxo Miocárdico, Stents Farmacológicos

A avaliação fisiológica intracoronária é estabelecida como uma estratégia valiosa para identificar estenoses epicárdicas limitantes de fluxo em pacientes com doença arterial coronariana (DAC) e para determinar uma indicação para intervenções coronárias percutâneas (ICP).^1^

Supunha-se anteriormente que, uma vez que a doença limitante do fluxo fosse confirmada por uma medida com fio-guia de pressão, a ICP guiada por angiografia deveria levar à restauração efetiva da condutância do vaso. No entanto, estudos com avaliação fisiológica pós-ICP com base na reserva de fluxo fracionada (FFR) e razões de pressão não hiperêmicas (NHPR) demonstraram que essa suposição não é correta e que confiar apenas na orientação angiográfica pode estar associado a resultados funcionais abaixo do ideal após a ICP em muitos casos.^2 , 3^ Além disso, sabe-se que a FFR pós-ICP é um forte preditor de desfechos, e quanto menor a FFR pós-stent, pior a evolução clínica.^4^

O artigo publicado por Pellegrini et al.,^5^ envolvendo 218 pacientes com DAC acompanhados por até 5 anos e submetidos à avaliação da FFR, mostrou maior número de MACE no grupo isquêmico tratado por ICP com stents farmacológicos (SF), em comparação com os grupos FFR normal baixa e FFR normal alta, sem diferenças entre estes dois últimos.

No entanto, algumas considerações precisam ser feitas a respeito deste estudo. Em primeiro lugar, trata-se de um estudo observacional, não randomizado, o que já implica inúmeras limitações, acrescidas pelo tamanho limitado da amostra, conforme apontado pelos autores na discussão das limitações do estudo. Em segundo lugar, o maior número de MACE no grupo isquêmico deveu-se à necessidade de nova revascularização do vaso-alvo, sem diferenças entre infarto e mortalidade entre os grupos, bem como sem diferenças entre o grupo ICP e o grupo FFR normal baixa. Estudos recentes, como o ensaio ISCHEMIA,^6^ não demonstraram benefício no tratamento de lesões estáveis em síndromes coronarianas crônicas, mesmo com SF, em comparação com o tratamento clínico otimizado. Além disso, os autores não informaram o valor médio da FFR pós-stent no grupo de tratamento, o que tem um impacto importante nos resultados pós-intervenção, conforme mencionado acima.

Terceiro, outra observação importante diz respeito ao número de casos tratados envolvendo a artéria descendente anterior (ADA), que é muito maior no grupo isquêmico (85,5%) do que nos outros dois grupos (65,9% e 43,1%, p < 0,001). A intervenção coronária percutânea em uma lesão na ADA foi previamente identificada como um preditor independente de resultados subótimos de FFR pós-ICP,^7 - 9^ e é crucial saber se a ADA foi responsável por uma pior FFR pós-stent, o que poderia explicar o maior número de MACE neste grupo.

Em quarto lugar, a FFR foi medida e um limiar ≤ 0,80 foi usado para tratar uma lesão. Curiosamente, o estudo IRIS^10^ e uma metanálise recente,^11^ ambos envolvendo mais de 6.000 lesões, demonstraram que um limiar de FFR ≤ 0,75 está associado a melhores resultados após a intervenção e que o risco de eventos adversos em lesões com FFR > 0,75 não foi significativamente diferente entre lesões deferidas e revascularizadas. Portanto, é possível que, se um limiar de FFR mais baixo fosse usado neste estudo, mais lesões teriam sido deferidas, o que geralmente está associado a melhores resultados.^12 , 13^

Por fim, concluímos que o estudo de Pellegrini et al.,^5^ levanta muito mais questões do que fornece respostas. A questão permanece: não deveríamos voltar a usar o valor de corte da FFR de 0,75 para indicar o tratamento de uma lesão coronariana estável, mesmo na era dos SF?
