# Supplementary material for: É Hora de Revisitar os Limites da Reserva de Fluxo Fracionada?
Source: Arq Bras Cardiol. 2023 Jun 28;120(6):e20230363. [Article in Portuguese] doi: 10.36660/abc.20230363 (PMC10365011; doi:10.36660/abc.20230363)
Supplement: Translation

# Translation: Is it Time to Revisit Fractional Flow Reserve Thresholds?

**Authors:** Fernando Mendes Sant’Anna, Lucas Bonacossa Sant’Anna, Sérgio Lívio Menezes Couceiro

**Affiliations:** 1 Brazil Universidade Federal do Rio de Janeiro - Campus Macaé, Macaé, RJ – Brazil; 2 Brazil Serviço de Hemodinâmica do Hospital Santa Izabel, Cabo Frio, RJ – Brazil; 3 Brazil Fundação Técnico-Educacional Souza Marques (FTESM), Rio de Janeiro, RJ – Brazil; 4 Brazil Departamento de Cardiologia do Hospital Santa Izabel, Cabo Frio, RJ – Brazil

**Keywords:** Pericardium, Constriction Pathoçogic, Coronary Artery Disease, Percutaneous Coronary Intervention, Fractional Flow Reserve, Myocardial, Drug Eluting Stents

Intracoronary physiological assessment is established as a valuable strategy to identify flow-limiting epicardial stenoses in patients with coronary artery disease (CAD) and to determine an indication for percutaneous coronary interventions (PCI).^1^

It had been previously assumed that once the flow-limiting disease was confirmed with a pressure guidewire measurement, PCI guided by angiography should lead to effective restoration of vessel conductance. However, studies with physiological post-PCI evaluation based on fractional flow reserve (FFR) and non-hyperemic pressure ratios (NHPR) demonstrate that this supposition is not correct and that relying on angiographic guidance alone can be associated with suboptimal functional results post-PCI in many cases.^2 , 3^ Moreover, it is well known that post-PCI FFR is a strong predictor of outcomes, and the lower the post-stent FFR, the worse the clinical follow-up.^4^

The article published by Pellegrini et al.,^5^ involving 218 patients with CAD followed for up to 5 years and submitted to FFR evaluation, showed a greater number of MACE in the ischemic group treated by PCI with drug-eluting stents (DES), compared to the low-normal FFR and high-normal FFR groups, with no differences between these two lasts.

However, some considerations need to be made concerning this study. First, it is an observational, non-randomized study, which already implies numerous limitations, increased due to the limited sample size, as pointed out by the authors in the discussion of the study’s limitations. Second, the greater number of MACE in the ischemic group was due to the need for new revascularization of the target vessel, with no differences between infarction and mortality between groups, as well as no differences between the PCI group and low-normal FFR group. Recent studies, such as the ISCHEMIA trial,^6^ have shown no benefit in treating stable lesions in chronic coronary syndromes, even with DES, compared with optimized clinical treatment. In addition, the authors did not inform the mean post-stent FFR value in the treatment group, which has an important impact on post-intervention results, as mentioned above.

Third, another important observation concerns the number of treated cases involving the left anterior descending artery (LAD), which is much higher in the ischemic group (85.5%) than in the two other groups (65.9% and 43.1%, p < 0.001). Percutaneous coronary intervention on a lesion in the LAD has previously been identified as an independent predictor of suboptimal post-PCI FFR results,^7 - 9^ and it is crucial to know if LAD was responsible for a worse post-stent FFR, which could explain the greater number of MACE is this group.

Fourth, FFR was measured, and a threshold ≤ 0.80 was used for treating a lesion. Interestingly, the IRIS trial^10^ and a recent meta-analysis,^11^ both involving more than 6,000 lesions, have demonstrated an FFR threshold of ≤ 0.75 to be associated with improved outcomes after intervention and that the risk of adverse events in lesions with FFR > 0.75 was not significantly different between deferred and revascularized lesions. Therefore, it is possible that if a lower FFR threshold were used in this study, more lesions would have been deferred, which is generally associated with better outcomes.^12 , 13^

Finally, we conclude that the study by Pellegrini et al.^5^ raises far more questions than it provides answers. The question remains: should we not return to using the FFR cutoff value of 0.75 to indicate treatment of a stable coronary lesion, even in the DES era?
